# Distribution of adenylyl cyclase/cAMP phosphodiesterase gene, *CAPE*, in streptophytes reproducing via motile sperm

**DOI:** 10.1038/s41598-021-89539-z

**Published:** 2021-05-12

**Authors:** Chiaki Yamamoto, Fumio Takahashi, Yosuke Ooe, Haruto Shirahata, Aika Shibata, Masahiro Kasahara

**Affiliations:** 1 Graduate School of Life Sciences, Ritsumeikan University, Shiga, 525-8577 Japan; 2 Department of Life Sciences, Ritsumeikan University, Shiga, 525-8577 Japan

**Keywords:** Plant evolution, Plant signalling

## Abstract

We recently isolated a novel adenylyl cyclase/cAMP phosphodiesterase gene from the liverwort, *Marchantia polymorpha*. The protein encoded by this gene has a class III adenylyl cyclase (AC) in the C-terminal domain and class I phosphodiesterase (PDE) in the N-terminal domain; therefore, we named it CAPE (COMBINED AC with PDE). CAPE protein is likely involved in spermatogenesis and sperm motility due to its tissue-specific expression pattern in *M. polymorpha* and the distribution of *CAPE* genes in streptophytes. However, little is known about the distribution of *CAPE* in gymnosperms that use motile sperm for fertilization, such as cycads and ginkgo. The present study aimed to isolate *CAPE* genes from the cycad, *Cycas revoluta*, the ginkgo, *Ginkgo biloba*, and the hornwort, *Anthoceros agerestis*. Sequences with high homology to *CAPE* were obtained from these species. Our analyses revealed that all plant taxonomic groups reproducing via motile sperm possessed *CAPE*, whereas those that do not produce motile sperm did not possess *CAPE*, with one exception in gymnosperm Cupressales. The phylogenic distribution of *CAPE* almost corresponds to the evolutionary history of motile sperm production and further suggests that *CAPE* may be involved in sexual reproduction process using motile sperm in streptophytes.

## Introduction

Adenosine 3',5'-cyclic monophosphate (cAMP) is a signaling molecule that regulates many biological activities in various organisms. It is synthesized from ATP by adenylyl cyclase (AC) and hydrolyzed to AMP by cAMP phosphodiesterase (PDE) to inactivate its activity as a signaling molecule. Intracellular cAMP levels are strictly regulated by the balance of AC and PDE activities^1^. ACs are classified according to their features into six classes (I–VI), which are evolutionarily unrelated to each other despite their common catalytic activity^2,3^. While class I, II, IV, V, and VI ACs are only present in a limited group of bacteria, most ACs belong to class III, which are widely distributed in prokaryotes and eukaryotes^4^. Guanylyl cyclases (GCs), which catalyze guanosine 3',5'-cyclic monophosphate (cGMP) synthesis, are homologous to class III ACs and emerged from a common ancestor^5,6^.

PDEs are classified into three classes according to the homology in the primary structure of their catalytic sites^1,4,7^. Class I PDEs are found in vertebrates and diverse eukaryotes. All mammalian PDEs belong to this class, which is subdivided into 11 families^1^. Class II PDEs are mainly found in yeasts and slime molds and class III comprises PDEs from bacteria^1,4^.

The physiological roles of cAMPs have been studied extensively in various organisms^4,8–10^. In prokaryotes, the cAMP receptor protein (CRP)–cAMP complex is involved in catabolite repression in *Escherichia coli*^11^. The CRP-like cAMP-activated global transcriptional regulator, GlxR, is a global regulator that controls the expression of various genes in *Corynebacterium glutamicum*^12^. cAMP signaling is required for virulence, and is used by *Bordetella pertussis* that causes whooping cough^13^, *Bacillus anthracis* that causes anthrax^14^, *Mycobacterium tuberculosis* that causes tuberculosis^15^, and the opportunistic human pathogen, *Pseudomonas aeruginosa*^16^. In addition, in eukaryotes, cAMP is involved in various cellular functions including glycogen degradation^17^, sense of taste signaling^18^, and flagellar movement of sperm^19^ in mammals, fruiting body formation in *Dictyostelium discoideum*^20,21^, and cell division in yeast^22^. Furthermore, cAMP plays important roles in photosynthetic organisms, such as light signaling and cell motility in cyanobacteria^23,24^, zygote formation in *Chlamydomonas reinhardtii*^25^, and the photophobic response in *Euglena gracilis*^26^. Thus, the diverse functions of cAMP have been clarified in many organisms.

In land plants, no homologous sequences coding for ACs or PDEs have been identified from other organisms in the genomes of angiosperms such as *Arabidopsis thaliana*^27^, *Oryza sativa*^28^, and *Solanum lycopersicum*^29^, although novel ACs, which are not classified into the existing AC classes, have been reported^30–32^. However, progress in genome and transcriptome analyses of basal land plant lineages such as bryophytes and lycophytes^33–35^ led to the identification of a protein with class III AC and class I PDE sequences, named COMBINED AC with PDE (CAPE) due to the presence of these catalytic domains in a single protein^36^.

CAPE is composed of a PDE domain at the N-terminus and an AC domain at the C-terminus. Biochemical analyses using *Marchantia polymorpha* (Mp) CAPE confirmed that both domains have each enzyme activity^36^. Mp*CAPE* is specifically expressed in the spermatogenous cells of antheridia (male sexual organ), but not in vegetative organs such as gemmalings and gametophytic plant bodies (thalli). Furthermore, *CAPE*s were only found in streptophytes that use motile sperm as the male gamete, such as bryophytes and ferns, but not in the genome of angiosperms^36^. However, the distribution of *CAPE* in the gymnosperms has not yet been clarified, although *CAPE* has not been found in the genomes of two species of Pinaceae, *Pinus taeda* and *Picea abies*^36–38^. Gymnosperms are diverse in terms of the delivery system of sperm to the egg cell. For example, Gnetales and Coniferae use non-motile sperm cells directly delivered by pollen tubes, whereas Cycadales and *Ginkgo* use motile sperm cells with flagella to swim to the egg cell. Thus, it is important to know whether cycads and ginkgo have *CAPE*.

Although the complete genomes of cycads and ginkgo have not yet been elucidated, a large amount of genetic information, especially transcriptome data, is available in public databases for many streptophytes including cycads and ginkgo. In our previous study, we were unable to identify the distribution of *CAPE* in taxa gymnosperms and the bryophyte, hornwort. Furthermore, the complete genomes from one gnetophyte, *Gnetum montanum*, and two zygnematophytes, *Spirogloea muscicola* and *Mesotaenium endlicherianum*, have recently been elucidated^39,40^. Thus, the present study investigated the distribution of *CAPE* in such streptophyte taxa in more detail.

## Results

### Database search of *CAPE* orthologs

We aimed to detect *CAPE* orthologs in some species of streptophyte lineage deposited in public databases using homology searches with the CAPE amino acid sequence from *M. polymorpha* (MpCAPE) as a query using the BLAST program.

### *C. revoluta* (cycad) and *G. biloba* (ginkgo)

We first focused on the distribution of *CAPE* in cycad and ginkgo because we previously reported that the distribution of *CAPE* coincided with that of the taxonomic groups that use motile sperms as male gamete in streptophytes^36^. A BLAST search was performed against RNA-seq data deposited in the sequence read archive (SRA) of GenBank. A large number of short sequences (reads), which were predicted to encode CAPE, were detected from the cycad and ginkgo RNA-seq data in the SRA.

### *A. agrestis* (hornwort)

The CAPE sequences in the liverwort, *M. polymorpha*, and the moss, *Physcomitrella patens*, were previously reported^36^. We were interested in the existence of *CAPE* in hornwort, the remaining taxonomic group in bryophytes for which the presence of *CAPE* has not been investigated. A BLAST search was performed against the genome DNA sequence data of *A. agrestis* in the SRA of GenBank^41,42^. Many short sequences were found that were predicted to encode CAPE.

### Zygnematophyceae and *Mesostigma viridae*

We reported that *CAPE* was not found from Zygnematophyceae and *M. viridae* following a search of the public RNA-seq databases^36^. Recently, complete genomes have been elucidated from two species in Zygnematophyceae, *Spirogloea muscicola* and *Mesotaenium endlicherianum*^40^, and *Mesostigma viridae*^43^. However, *CAPE* was not detected in these genomes.

### Gymnosperms other than cycad and ginkgo

We reported that *CAPE* was not detected in the genomes of two species of Pinaceae, *Pinus taeda* and *Picea abies*^36^. The complete genome of a gnetophyte, *Gnetum montanum*, was recently elucidated^39^. However, we were unable to detect *CAPE* in the genome of *G. montanum*. On the other hand, we searched for *CAPE* orthologs in other taxonomic groups of gymnosperms due to the high diversity of gymnosperms. First, a homology search was performed for OneKP, which is large-scale gene sequencing data mainly based on transcriptomes of > 1000 species of plants^44^. *CAPE*s were detected from streptophyte algae and bryophytes, in which the distribution of *CAPE* was reported in our previous study^36^. Unexpectedly, scaffold sequences, of which the deduced amino acid sequences exhibit significant homology to a part of Mp*CAPE*, were detected from gymnosperms that do not produce motile sperms, including four species each from Cupressaceae and Taxaceae (Table S3). In particular, a scaffold sequence of *Cephalotaxus harringtonia* (GJTI_scaffold_2061849) contained the sequence encoding both AC and PDE partial sequences. On the other hand, scaffolds exhibiting the homology to *CAPE* were not detected in any species of Pinaceae, which was consistent with a previous report that stated that *CAPE* was not detected in the genomes of *P. taeda* and *P. abies*^36^. In addition to searching the OneKP database, a BLAST search was performed against the RNA-seq data of a Cupressaceae, *Cryptomeria japonica*, deposited in the SRA of GenBank. Many short sequences were detected that were predicted to encode CAPE.

### Isolation of *CAPE* genes from *C. revoluta, G. biloba, C. japonica*, and *A. agrestis*

**Figure 1:**
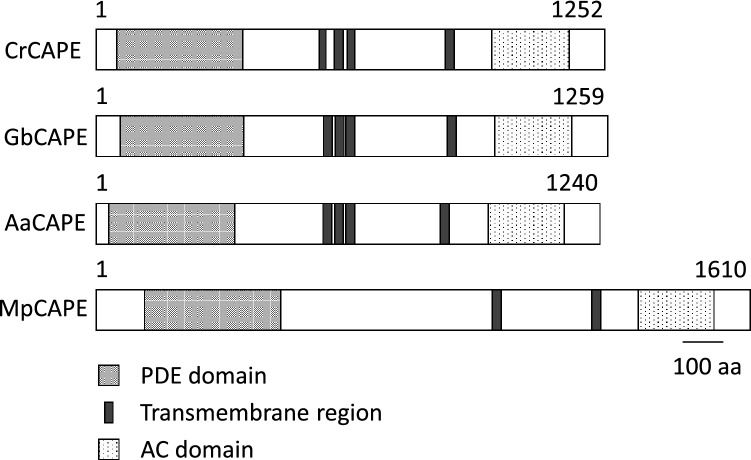
Schematic representation of the domain organization of CAPE. The catalytic domains of PDE and AC are shown as gray and boxes filled with dots, respectively. Membrane-spanning regions are shown as thin black boxes. Aa, *Anthoceros agrestis*; Cr, *Cycas revoluta*; Gb, *Ginkgo biloba*; Mp, *Marchantia polymorpha.*

We found partial sequences of CAPE-like genes from each RNA-seq data in the SRA. RT-PCR was performed to obtain the full-length coding sequence of *CAPE* using mRNA from *C. revoluta* (Cr), *G. biloba* (Gb), *A. agrestis* (Aa), and *C. japonica* (Cj), followed by 3′, 5′- RACE PCR for *C. revoluta* and *G. biloba*. The cDNAs containing the full-length CAPE coding sequences were obtained from *C. revoluta, G. biloba*, and *A. agrestis*. The deduced amino acid sequences of CrCAPE, GbCAPE, and AaCAPE were determined and comprised 1252 (CrCAPE), 1259 (GbCAPE), and 1240 (AaCAPE) amino acids, respectively, and contained one PDE domain at the N-terminus, multiple transmembrane regions located at the middle part of the protein, and one AC domain near the C-terminus (Fig. 1 and Fig. S1). Four transmembrane regions were predicted in CrCAPE, GbCAPE, and AaCAPE. The presence of multiple transmembrane regions in the middle part of CAPE appears to be a common feature of CAPE (Fig. S2). Two cDNA clones were obtained for *C. japonica*: Cj*CAPE* clone#1 and Cj*CAPE* clone#2. Cj*CAPE* clone#1 encoded a partial PDE domain that lacked 14 amino acids at the N-terminus and had a frameshift, causing different reading frames of PDE and AC (Fig. S3). Cj*CAPE* clone#2 encoded PDE domain and an incomplete AC domain, caused by an insertion of 42 bp at the middle of the AC-coding sequence (Fig. S3).

### Comparison of the AC domain of CAPE to the class III ACs

**Figure 2:**
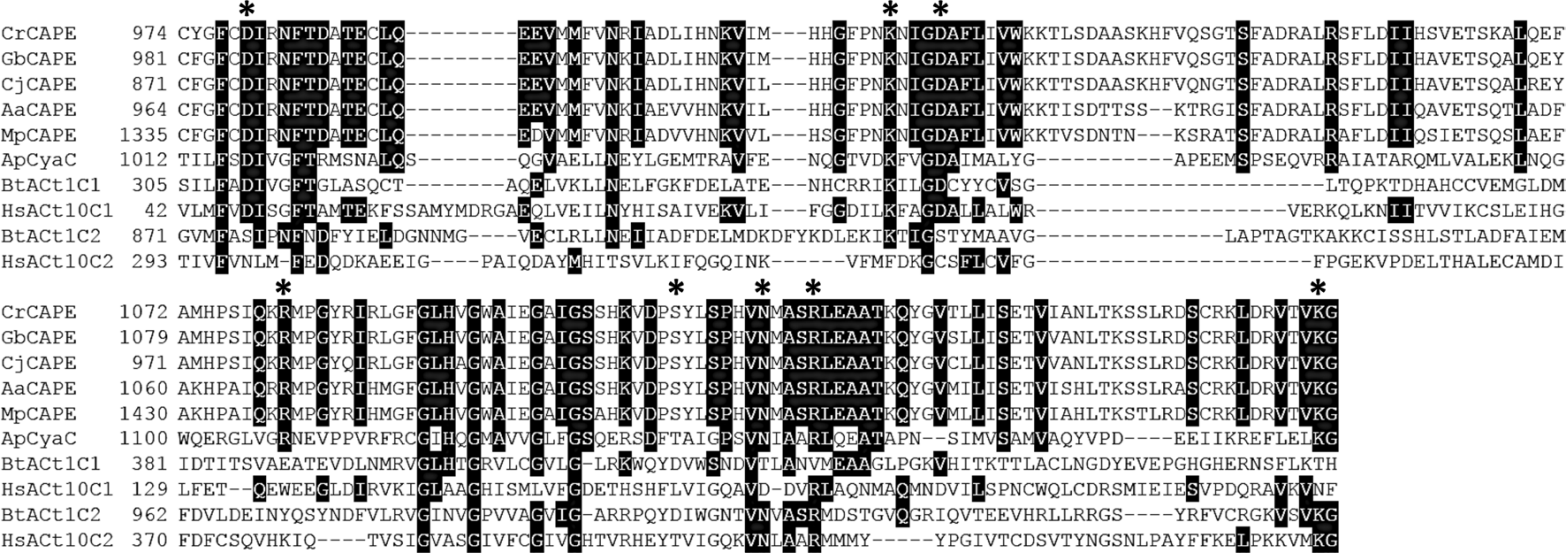
Amino acid alignment of the AC domain of CAPE. Multiple alignment of the AC domain of CAPE with *Arthrospira platensis* CyaC (ApCyaC), *Bos taurus* type 1 AC (BtACt1C1 and BtACt1C2), and *Homo sapiens* type 10 AC (HsACt10C1 and HsACt10C2). Amino acids involved in binding the substrate ATP are indicated by asterisks. Amino acid residues that were identical in the majority of the sequences are shown in black. Aa, *Anthoceros agrestis*; Cj, *Cryptomeria japonica*; Cr, *Cycas revoluta*; Gb, *Ginkgo biloba*; Mp, *Marchantia polymorpha.*

The amino acid sequences of the AC domains of Cr, Gb, Cj, and AaCAPE were compared with the class III ACs, MpCAPE-AC, *Arthrospira platensis* CyaC (ApCyaC), *Bos taurus* type 1 AC (BtACt1C1 and BtACt1C2), and *Homo sapiens* type 10 AC (HsACt10C1 and HsACt10C2). The eight consensus amino acids required for catalysis and substrate binding were completely conserved in the AC domains of CrCAPE, GbCAPE, and AaCAPE (Fig. 2). The match rates of each AC domains to the amino acid sequence of MpCAPE-AC domain were 83% identity and 96% similarity for CrCAPE, 82% identity and 96% similarity for GbCAPE, 82% identity and 96% similarity for CjCAPE (clone#1), and 87% identity and 98% similarity for AaCAPE.

### Comparison between CAPE PDE domain and class I PDEs

**Figure 3:**
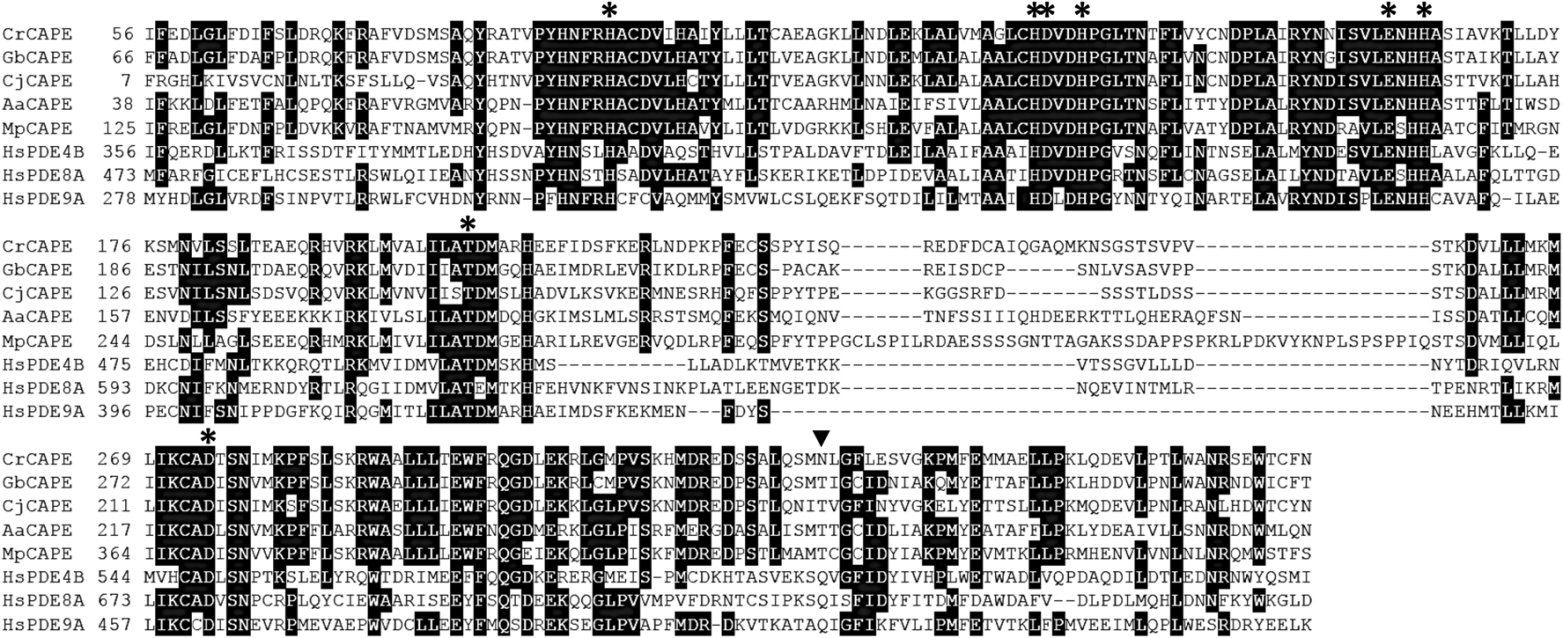
Amino acid alignment of the PDE domain of CAPE. Multiple alignment of the PDE domain of CAPE with *Homo sapiens* PDE4B, PDE8A, and PDE9A. Amino acids involved in metal ion binding are indicated by asterisks. Arrowhead shows amino acid residues involved in the recognition of purine bases. Amino acid residues found to be identical in the majority of the sequences are shown in black. Aa, *Anthoceros agrestis*; Cj, *Cryptomeria japonica*; Cr, *Cycas revoluta*; Gb, *Ginkgo biloba*; Mp, *Marchantia polymorpha.*

The amino acid sequences of the PDE domains of Cr, Gb, Cj, and Aa CAPE were compared with class I PDEs, MpCAPE-PDE, *Homo sapiens* PDE4B, PDE8A, and PDE9A. In PDEs, two divalent metal cations are coordinated by highly conserved amino acids in the catalytic domain and are essential for catalysis. The PDE domains of isolated CAPEs completely retained the eight amino acid residues necessary for the metal binding (Fig. 3). In class I PDEs, purine bases (A or G) of the substrates (ATP or GTP) are recognized by the highly conserved glutamine (Fig. 3), whereas the corresponding amino acid residues in the PDE domains of CrCAPE and CjCAPE are replaced by asparagine, and those of GbCAPE, AaCAPE, and MpCAPE are replaced by threonine (Fig. 3). The match rates of each domain to the amino acid sequence of MpCAPE-PDE domain were 49% identity and 77% similarity for CrCAPE, 54% identity and 78% similarity in GbCAPE, 46% identity and 74% similarity for CjCAPE (clone#2), 48% identity and 80% similarity for AaCAPE.

### Phylogenetic analyses of the class III AC and class I PDE catalytic domains

**Figure 4:**
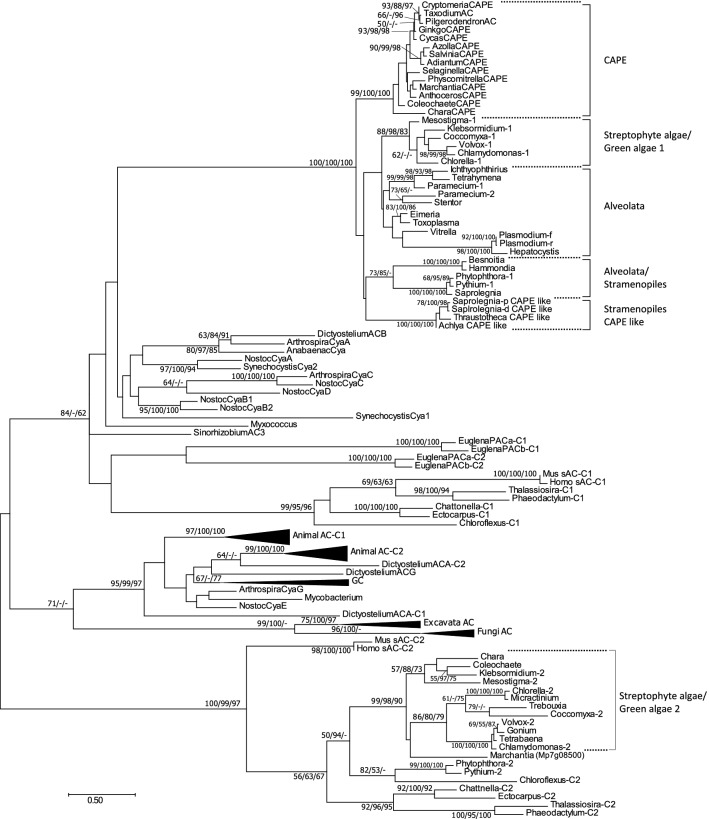
Phylogenetic tree of catalytic domains of class III ACs and GCs. The phylogenetic tree was inferred using the maximum likelihood method with the LG + G model. All positions with < 70% site coverage were eliminated. Numbers represent support values (> 50%) obtained with 100 bootstrap replicates using the MEGA7 software (LG + G mode). Numerical numbers before branch nodes are ML, NJ, and MP. The evolutionary distances were calculated in units of the number of amino acid substitutions per site as indicated by the scale bar below the tree. The accession numbers of the AC and GC sequences used for the phylogenetic analysis are shown in Table S1.

Phylogenetic analysis was performed using the amino acid sequences of the catalytic domains of the CAPE-ACs and the class III ACs and GCs (Fig. 4). The CAPE-ACs formed a monophyletic group in which the tree topology among CAPE-ACs was consistent with the phylogeny in streptophytes. The CAPE-ACs were found to be closely related to those of Streptophyte algae, Green algae, Alveolata and Stramenopiles ACs (Fig. 4). Apart from the group (Streptophyte algae/Green algae 1) closely related to CAPE, another group of green plant AC-related sequences (Streptophyte algae/Green algae 2) was formed. Another AC-like sequence (Mp7g08500.1) that differed to CAPE was present in the *M. polymorpha* genome^36^ and formed a group with Streptophyte algae/Green algae 2.

**Figure 5:**
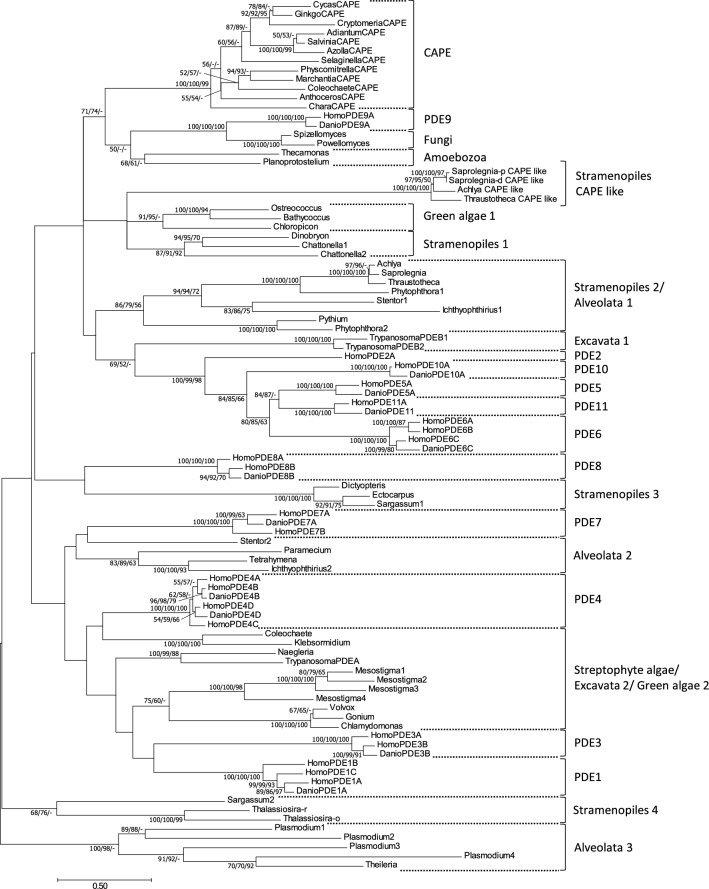
Phylogenetic tree of catalytic domains of class I PDE. The phylogenetic tree was inferred using the maximum-likelihood method with the LG + G + I model. All positions containing gaps and missing data were eliminated. Numbers represent support values (> 50%) obtained with 100 bootstrap replicates using the MEGA7 software (LG + G + I mode). Numerical numbers before the branch nodes are ML, NJ, and MP. The evolutionary distances were computed in units of the number of amino acid substitutions per site as indicated by the scale bar below the tree. The accession numbers of PDE sequences used for the phylogenetic analysis are shown in Table S2.

Phylogenetic analysis was performed with the amino acid sequences of the catalytic domains of the CAPE-PDEs and class I PDEs (Fig. 5). The CAPE-PDEs formed a monophyletic group in which the tree topology among CAPE-PDEs was almost consistent with the phylogeny in streptophytes. The catalytic domain of CAPE-PDEs was found to be closely related to the animal PDE9 family (Fig. 5).

## Discussion

The present study elucidated more precise information about the distribution of *CAPE* in streptophytes by isolating its orthologs from gymnosperms, *C. revoluta*, *G. biloba*, *C. japonica*, and the hornwort, *A. agrestis*. The distribution of *CAPE* genes is summarized in Table 1, which includes information about the taxonomic groups that produce motile sperm in streptophytes. All streptophytes that reproduce with motile sperm, including *C. revoluta* and *G. biloba*, retained the *CAPE* genes. In streptophytes, *CAPE* is thought to have emerged during the evolutionary process between Klebsormidiophyceae and Charophyceae^36^. Charophyceae were the first streptophytes to develop motile sperm, and the architecture of the sperm is very similar to that of the basal land plants^45^. Thus, the appearance of *CAPE* in *Chara braunii* coincides with the emergence of motile sperm as the male gamete in streptophytes. On the other hand, *CAPE* was lost multiple times, each independently, during the formation of streptophytes taxa including Zygonematophyceae, parts of gymnosperms (Gnetaceae and Pinaceae), and angiosperms. Therefore, CAPE may play an important role in motile sperm function. The role of cAMP in the regulation of flagellar motility has been investigated in mammals and algae^46,47^, and it has been suggested that cAMP may also be involved in motile sperm regulation in plants. The role of cAMP in plant cells may be restricted to the function of motile sperm, and it is hypothesized that loss of the motile sperm system coincides with loss of *CAPE*. On the other hand, *CAPE* is present in gymnosperms that do not produce motile sperms such as *C. japonica*.

**Table 1:** Distribution of *CAPE* in streptophytes.

Group	Subgroup	Species	Motile sperm^a^	*CAPE*^b^
Angiosperms		*Arabidopsis thaliana*		△
		*Oryza sativa*		△
		*Solanum lycopersicum*		△
Gymnosperms	Cupressaceae	*Cryptomeria japonica*		●
	Pinaceae	*Pinus taeda*		△
		*Picea abies*		△
	Gnetaceae	*Gnetum montanum*		△
	Cycadaceae	*Cycas revoluta*	●	●
	Ginkgoaceae	*Ginkgo biloba*	●	●
Ferns		*Adiantum capillus-veneris*	●	●
Lycophytes		*Selaginella moellendorffii *	●	●
Bryophytes	Mosses	*Physcomitrella patens*	●	●
	Liverworts	*Marchantia polymorpha*	●	●
	Hornworts	*Anthoceros agrestis*	●	●
Streptophyte algae	Zygnematophyceae	*Spirogloea muscicola*		△
		*Mesotaenium endlichaerianum*		△
	Coleochaetaceae	*Coleochaete orbicularis *	●	●
	Characeae	*Chara braunii *	●	●
	Klebsormidiaceae	*Klebsormidium flaccidum *		△
	Mesostigmataceae	*Mesostigma viride*		△
Chlorophyte algae		*Chlamydomonas reinhardtii *		△
		*Chlorella variabilis*		△

^a^Species reproducing with motile sperm are marked by black circles (●).

^b^Species having *CAPE* gene are marked black circles (●). Species, in which *CAPE* gene were not detected in the complete genomes, are marked by triangles (△).

In the present study, two cDNA clones were obtained from *C. japonica*: Cj*CAPE* clone#1 and Cj*CAPE* clone#2 (Fig. S3). The presence of the cDNA clones containing both PDE and AC coding sequences indicated that *C. japonica* contains the *CAPE* gene within its genome. In mammals, PDE families contain many splice variants that are related to the regulation of cellular signaling pathways^48–50^, and multiple transcripts from *CAPE* gene are registered in the *M. polymorpha* genome database as a result of alternative splicing. It is likely that mRNA encoding both PDE and AC domains in one protein is expressed in the specific stages of the life cycle or tissues of *C. japonica*. Furthermore, in OneKP database, putative *CAPE* sequences in Cupressales were detected in four species of Cupressaceae (*Callitris gracilis*, *Pilgerodendron uviferum*, *Taxodium distichum*, and *Widdrigntonia cedarbergensis*) and four species of Taxaceae (*Amentotaxus argotaenia*, *Cephalotaxus harringtoina*, *Pseudotaxus chienii*, and *Torreya uncifera*) (Table S3). This wide distribution in Cupressales is likely to indicate that *CAPE* is conserved in Cupressales rather than being maintained in only a few species of Cupressales. Investigating *CAPE* in Cupressales may help to elucidate the function of cAMP other than that related to motile sperm.

The AC domains of CrCAPE, GbCAPE, CjCAPE, and AaCAPE had fully eight conserved consensus amino acids required for catalysis and substrate binding, and high match rates to MpCAPE (Fig. 2). MpCAPE has been shown to have adenylyl cyclase activity^36^; therefore, it is predicted that they will have cAMP synthesis activity. The PDE domains of CrCAPE, GbCAPE, CjCAPE, and AaCAPE also retained all eight amino acids required for metal binding and showed high match rates to MpCAPE (Fig. 3). The glutamine residue, which is one of the most conserved amino acids among the class I PDEs and is involved in the recognition of purine base of the substrate, was replaced by asparagine in CrCAPE and CjCAPE-PDEs, and by threonine in GbCAPE, AaCAPE, and MpCAPE-PDEs (Fig. 3). CrCAPE and CjCAPE are predicted to have PDE activity since glutamine and asparagine have similar amino acid properties. Furthermore, MpCAPE-PDE has been shown to have PDE activity^36^; thus GbCAPE-PDE and AaCAPE-PDE are also expected to have PDE activity. In summary, the AC and PDE domains of CAPE should have cAMP synthesis/hydrolysis activity, respectively, and it is expected that cAMP functions as a signaling molecule in plants expressing *CAPE*.

Most CAPEs have an even number of transmembrane sites between the PDE and AC domains (Fig. S2). CAPEs should be localized to the membrane and the PDE and AC domains are likely to face the same side of the membrane. The cellular level of cAMP can be strictly controlled via cAMP synthesis and hydrolysis by the AC and PDE domains of CAPE, respectively. Different cAMP effectors regulate each specific signaling process in the cytoplasm; thus, cAMP must be prevented from free diffusion and localized in small restricted areas to activate specific effectors^51,52^. CAPEs may facilitate the spatial regulation of cAMP by having both domains in the cytoplasmic region.

Phylogenetic analysis of AC revealed that CAPE-AC was closely related to streptophyte and green algal AC (Fig. 4; streptophyte algae/green algae 1). A chimeric protein may be produced by the fusion of streptophyte algae/green algae 1 AC and PDE, as an ancestor of CAPE. After PDE fusion, streptophyte algae/green algae 1 type AC and CAPE-AC appeared to be phylogenetically separated from their common AC ancestor. On the other hand, there were no green algal PDEs that showed a close relationship with CAPE-PDE, although animal PDE9, and fungal and amoebozoal PDEs had a close relationship with CAPE-PDE (Fig. 5). Therefore, CAPE-PDE may have a common ancestor with these PDEs. However, the positions of the animal PDEs (PDE1-PDE11) are scattered in the phylogenetic tree, and the animal PDEs do not form a monophyletic group, suggesting that PDEs may have fast molecular evolutionary rates to optimize their function. Therefore, it is possible that CAPE-PDE may have convergently evolved from an ancestral PDE of streptophyte algae and may have become similar to PDE9.

Phylogenetic analysis of PDE revealed that CAPE-PDE was closely related to the animal PDE9 family (Fig. 5). PDE9 is a cGMP-specific enzyme and has the lowest *Km* of any PDE for cGMP in all PDE families^49,53^. Although MpCAPE-PDE is cAMP-specific^36^, it could have a low *Km* for cAMP. MpCAPE-AC activity is relatively low compared with that of other class III ACs^36^, making it a high affinity type to effectively degrade cAMP.

Phylogenetic analyses of the class III AC and class I PDE sequences revealed that genes encode proteins with the same domain organizations as CAPE, that is, proteins containing both class III AC and class I PDE domains in non-photosynthetic stramenopiles, *Saprolegnia diclina*, *Saprolegnia parasitica*, *Achlya hypogyna*, and *Thraustotheca clavata*. Phylogenetic analyses demonstrated that both domains in non-photosynthetic stramenopile CAPE-like sequences were separated from those in plant CAPEs, indicating that they are produced independently. These CAPE-like proteins may play a role in cAMP signaling in non-photosynthetic stramenopiles.

We found that *CAPE* was conserved in plants that reproduce with sperm (Table 1). Among land plants, cycads, ginkgo, ferns, lycophytes, and bryophytes, which produce sperm, all retained *CAPE*. Even in streptophyte algae, *CAPE* is present in Coleochaetaceae and Characeae, which produce motile sperm, but not in Klebsormidiaceae and Zygnematophyceae, which do not produce sperm. The distribution of *CAPE* in streptophytes (Table 1) and the specific expression of Mp*CAPE* in spermatogenous cells of antheridia^36^ strongly suggest that CAPE plays a role in motile sperm function. Analysis of *CAPE* mutants constructed by gene targeting in model plants, such as *M. polymorpha* and *P. patens*, could verify this and clarify the physiological role of cAMP in plants.

## Methods

### Database search

We used the amino acid sequence of *M. polymorpha* CAPE to perform a BLAST search using the public NCBI website and the tblastn program against RNA-seq data for *Cycas revoluta* (SRX661923), *Ginkgo biloba* (SRX1135298, SRX1135299, SRX1135300, and SRX1084991), or *Cryptomeria japonica* (DRX001291-001294, DRX081263-081272, DRX155737, DRX155749, and DRX155777). For *Anthoceros agrestis*, its whole genome shotgun sequence data ERX714368 and ERX714369^42^ were assembled with pipeline at DNA Data Bank of Japan (DDBJ) and the assembled contig data by tblastn program was searched using *M. polymorpha* CAPE sequence as a query. Polymerase chain reaction (PCR) primers were designed using the retrieved sequences. A BLAST search was performed at the public OneKP website using the tblastn program using the amino acid sequence of *M. polymorpha* CAPE to obtain the partial sequences of CAPE in gymnosperms.

### Preparation of total RNA from *C. revoluta, G. biloba, C. japonica*, and *A. agrestis*

RNA samples were prepared from ovules harvested at the time when sperm cells were developing in pollinated pollen grains from *C. revoluta* or *G. biloba*, male strobili of *C. japonica*, and gametophytes bearing antheridia of *A. agrestis* (Oxford isolate) using the cetyltrimethylammonium bromide (CTAB) method^54^. Frozen specimens were broken up using a mortar and pestle with quartz sand in liquid nitrogen and then incubated in CTAB solution (2% CTAB, 1.4 M NaCl, 0.2% 2-mercaptoethanol, 20 mM EDTA, and 100 mM Tris–HCl, pH 8.0) at 60 ˚C for 30 min. RNA was prepared from *A. agrestis* and *C. japonica* using CTAB solution supplemented with 1% polyvinylpyrrolidone-40. RNA extraction was performed three times using chloroform and precipitated with 2-propanol. The precipitates were washed with 70% ethanol and dissolved in RNase-free water. RNA was precipitated again with 2 M LiCl overnight on ice. The samples were centrifuged at 11,000 *g* for 30 min and the RNA was washed with 70% ethanol and dissolved in RNase-free water.

### Isolation of *CAPE* cDNAs from *C. revoluta* and *G. biloba* by reverse transcriptase (RT)-PCR and 5' and 3'-RACE

*C. revoluta* and *G. biloba CAPE* cDNA samples were generated by RT-PCR using total RNA samples with the following primers: CrCAPE-f, 5'-ATTTGCCAAAGATGGTGGAG-3' and CrCAPE-r, 5'-TCCTATGGGAAGCCATGAAG-3' for *C. revoluta* and GbCAPE-f, 5'-GCATTTCCCCTCGATAGACA-3' and GbCAPE-r, 5'-TGAATGCAGACAATCAGGGA-3' for *G. biloba*. Amplified PCR products were cloned into pCR-Blunt II-TOPO vectors (Thermo Fisher, MA, USA) and the nucleotide sequences were determined. Since the 5' and 3' ends for *C. revoluta* and the 5' end for *G. biloba* were not contained in the PCR products, 5'- and 3'-RACE was performed to obtain the terminal fragments using 5'- and 3'-RACE kits (Thermo Fisher). Finally, full-length cDNA fragments were amplified by RT-PCR using primers designed using the sequences encoding the initiation and termination codons, cloned into a low-copy plasmid, pSTV29 (Takara Bio, Shiga, Japan), and sequenced to verify the full-length sequences.

### Isolation of *CAPE* cDNAs from* A. agrestis* and *C. japonica* by RT-PCR

*A. agrestis CAPE* cDNA was amplified by RT-PCR using total RNA samples with the following primers: AaCAPE-f, 5'-CACCATGGCCAATTTTGATGAGGATG-3' and AaCAPE-r, 5'-TCATTTTTCTGTTAATTCACGATAC-3'. The amplified PCR products were cloned into pENTR/D-TOPO (Thermo Fisher) and sequenced. *C. japonica CAPE* cDNAs were generated RT-PCR using total RNA samples with the following primers: CjCAPE-f1, 5'-GGCATGCCTGTGATGTCTTA-3' for clone#1 or CjCAPE-f2, 5'-ATAGTAGATTCACCTCCATTTAG-3' for clone#2 and CjCAPE-r, 5'-TCATTTCTCTGTTAGTTCCCTGTATCC-3'. Amplified PCR products were cloned into pCR-Blunt II-TOPO vectors (Thermo Fisher) and the nucleotide sequences were determined.

### Phylogenic analysis of CAPE

The amino acid sequences of the AC and PDE domains (Tables S1 and S2) were aligned using ClustalX2.0^55^. The maximum likelihood (ML) model of AC and PDE selected by the model test of MEGA 7.0^56^. ML, the neighbor joining (NJ) method, and most-parsimonious (MP) were performed using bootstrap values.

## Supplementary Information

Supplementary Information.

## References

[1] Conti M, Beavo J (2007). Biochemistry and physiology of cyclic nucleotide phosphodiesterases: Essential components in cyclic nucleotide signaling. Annu. Rev. Biochem..

[2] Danchin A (1993). Phylogeny of adenylyl cyclases. Adv. Second Messenger Phosphoprotein Res..

[3] Linder JU, Schultz JE (2003). The class III adenylyl cyclases: Multi-purpose signalling modules. Cell Signal..

[4] Gancedo JM (2013). Biological roles of cAMP: Variations on a theme in the different kingdoms of life. Biol. Rev. Camb. Philos. Soc..

[5] Tucker CL, Hurley JH, Miller TR, Hurley JB (1998). Two amino acid substitutions convert a guanylyl cyclase, RetGC-1, into an adenylyl cyclase. Proc. Natl. Acad. Sci. U S A..

[6] Tesmer JJ (1999). Two-metal-ion catalysis in adenylyl cyclase. Science.

[7] Richter W (2002). 3',5' Cyclic nucleotide phosphodiesterases class III: members, structure, and catalytic mechanism. Proteins.

[8] Botsford JL, Harman JG (1992). Cyclic AMP in prokaryotes. Microbiol. Rev..

[9] Dessauer, C. W. *et al.* International Union of Basic and Clinical Pharmacology. CI. Structures and small molecule modulators of mammalian adenylyl cyclases. *Pharmacol. Rev*. **69**, 93–139 (2017). 10.1124/pr.116.013078 PMC5394921 28255005

[10] Bassler J, Schultz JE, Lupas AN (2018). Adenylate cyclases: Receivers, transducers, and generators of signals. Cell Signal..

[11] Ullmann A, Monod J (1968). Cyclic AMP as an antagonist of catabolite repression in *Escherichia coli*. FEBS Lett..

[12] Schröder J, Tauch A (2010). Transcriptional regulation of gene expression in Corynebacterium glutamicum: The role of global, master and local regulators in the modular and hierarchical gene regulatory network. FEMS Microbiol. Rev..

[13] Hanski E (1989). Invasive adenylate cyclase toxin of *Bordetella pertussis*. Trends Biochem. Sci..

[14] Gordon, V. M. *et al.* Adenylate cyclase toxins from *Bacillus anthracis* and *Bordetella pertussis*. Different processes for interaction with and entry into target cells. *J. Biol. Chem*. **264**, 14792–14796 (1989). 2504710

[15] Agarwal N, Lamichhane G, Gupta R, Nolan S, Bishai WR (2009). Cyclic AMP intoxication of macrophages by a *Mycobacterium tuberculosis* adenylate cyclase. Nature.

[16] Fuchs EL (2010). In vitro and in vivo characterization of the *Pseudomonas aeruginosa* cyclic AMP (cAMP) phosphodiesterase CpdA, required for cAMP homeostasis and virulence factor regulation. J. Bacteriol..

[17] Sutherland EW (1972). Studies on the mechanism of hormone action. Science.

[18] Margolskee RF (2002). Molecular mechanisms of bitter and sweet taste transduction. J. Biol. Chem..

[19] Xie F (2006). Soluble adenylyl cyclase (sAC) is indispensable for sperm function and fertilization. Dev. Biol..

[20] Konijn TM, Van De Meene JG, Bonner JT, Barkley DS (1967). The acrasin activity of adenosine-3',5'-cyclic phosphate. Proc. Natl. Acad. Sci. U S A.

[21] Loomis WF (2014). Cell signaling during development of Dictyostelium. Dev. Biol..

[22] Matsumoto K, Uno I, Oshima Y, Ishikawa T (1982). Isolation and characterization of yeast mutants deficient in adenylate cyclase and cAMP-dependent protein kinase. Proc. Natl. Acad. Sci. U S A..

[23] Terauchi K, Ohmori M (2004). Blue light stimulates cyanobacterial motility via a cAMP signal transduction system. Mol. Microbiol..

[24] Ohmori M, Okamoto S (2004). Photoresponsive cAMP signal transduction in cyanobacteria. Photochem. Photobiol. Sci..

[25] Wang Q, Pan J, Snell WJ (2006). Intraflagellar transport particles participate directly in cilium-generated signaling in Chlamydomonas. Cell.

[26] Iseki M (2002). A blue-light-activated adenylyl cyclase mediates photoavoidance in *Euglena gracilis*. Nature.

[27] Initiative AG (2000). Analysis of the genome sequence of the flowering plant *Arabidopsis thaliana*. Nature.

[28] Project, I. R. G. S (2005). The map-based sequence of the rice genome. Nature.

[29] Consortium, T. G (2012). The tomato genome sequence provides insights into fleshy fruit evolution. Nature.

[30] Gehring C (2010). Adenyl cyclases and cAMP in plant signalling—Past and present. Cell Commun. Signal..

[31] Gehring C, Turek IS (2017). Cyclic nucleotide monophosphates and their cyclases in plant signaling. Front. Plant Sci..

[32] Chatukuta, P. *et al.* An Arabidopsis clathrin assembly protein with a predicted role in plant defense can function as an adenylate cyclase. *Biomolecules* **8** (2018). 10.3390/biom8020015 PMC6022867 29570675

[33] Rensing SA (2008). The Physcomitrella genome reveals evolutionary insights into the conquest of land by plants. Science.

[34] Banks JA (2011). The Selaginella genome identifies genetic changes associated with the evolution of vascular plants. Science.

[35] Bowman JL (2017). Insights into land plant evolution garnered from the *Marchantia polymorpha* genome. Cell.

[36] Kasahara M (2016). An adenylyl cyclase with a phosphodiesterase domain in basal plants with a motile sperm system. Sci. Rep..

[37] Nystedt B (2013). The Norway spruce genome sequence and conifer genome evolution. Nature.

[38] Neale DB (2014). Decoding the massive genome of loblolly pine using haploid DNA and novel assembly strategies. Genome Biol..

[39] Wan T (2018). A genome for gnetophytes and early evolution of seed plants. Nat. Plants..

[40] Cheng S (2019). Genomes of subaerial Zygnematophyceae provide insights into land plant evolution. Cell.

[41] Li FW (2020). Anthoceros genomes illuminate the origin of land plants and the unique biology of hornworts. Nat. Plants..

[42] Szövényi P (2015). Establishment of *Anthoceros agrestis* as a model species for studying the biology of hornworts. BMC Plant Biol..

[43] Wang S (2020). Genomes of early-diverging streptophyte algae shed light on plant terrestrialization. Nat Plants..

[44] Initiative OTPT (2019). One thousand plant transcriptomes and the phylogenomics of green plants. Nature.

[45] Renzaglia KS, Garbary DJ (2001). Motile gametes of land plants: Diversity, development, and evolution. Crit. Rev. Plant Sci..

[46] Tresguerres M, Levin LR, Buck J (2011). Intracellular cAMP signaling by soluble adenylyl cyclase. Kidney Int..

[47] Saegusa Y, Yoshimura K (2015). cAMP controls the balance of the propulsive forces generated by the two flagella of Chlamydomonas. Cytoskeleton (Hoboken)..

[48] Rentero C, Monfort A, Puigdomènech P (2003). Identification and distribution of different mRNA variants produced by differential splicing in the human phosphodiesterase 9A gene. Biochem. Biophys. Res. Commun..

[49] Bender AT, Beavo JA (2006). Cyclic nucleotide phosphodiesterases: Molecular regulation to clinical use. Pharmacol. Rev..

[50] Omori K, Kotera J (2007). Overview of PDEs and their regulation. Circ. Res..

[51] Baillie GS (2009). Compartmentalized signalling: Spatial regulation of cAMP by the action of compartmentalized phosphodiesterases. FEBS J..

[52] Conti M, Mika D, Richter W (2014). Cyclic AMP compartments and signaling specificity: Role of cyclic nucleotide phosphodiesterases. J. Gen. Physiol..

[53] Fisher, D. A., Smith, J. F., Pillar, J. S., St Denis, S. H. & Cheng, J. B. Isolation and characterization of PDE9A, a novel human cGMP-specific phosphodiesterase. *J. Biol. Chem*. **273**, 15559–15564 (1998). 10.1074/jbc.273.25.15559 9624146

[54] Imaizumi T, Kanegae T, Wada M (2000). Cryptochrome nucleocytoplasmic distribution and gene expression are regulated by light quality in the fern *Adiantum capillus-veneris*. Plant Cell.

[55] Larkin, M. A. *et al.* Clustal W and Clustal X version 2.0. *Bioinformatics*. **23**, 2947–2948 (2007). 10.1093/bioinformatics/btm404 17846036

[56] Kumar, S., Stecher, G. & Tamura, K. MEGA7: Molecular evolutionary genetics analysis version 7.0 for bigger datasets. *Mol. Biol. Evol*. **33**, 1870–1874 (2016). 10.1093/molbev/msw054 PMC8210823 27004904

