# Supplementary material for: Distribution of adenylyl cyclase/cAMP phosphodiesterase gene, CAPE, in streptophytes reproducing via motile sperm
Source: Sci Rep. 2021 May 12;11:10054. doi: 10.1038/s41598-021-89539-z (PMC8115329; doi:10.1038/s41598-021-89539-z)
Supplement: Supplementary file 1 — Supplementary Information. [file 41598_2021_89539_MOESM1_ESM.pdf]

Supplementary Information for

Distribution of adenylyl cyclase/cAMP phosphodiesterase gene, *CAPE*, in streptophytes reproducing via motile sperm

Chiaki Yamamoto[1], Fumio Takahashi[1], Yosuke Ooe[2], Haruto Shirahata[2], Aika Shibata[1], and Masahiro Kasahara[1,*]

[1]Graduate School of Life Sciences, Ritsumeikan University, Shiga 525-8577, Japan
[2]Department of Life Sciences, Ritsumeikan University, Shiga 525-8577, Japan

*To whom correspondence should be addressed: kasa@sk.ritsumei.ac.jp

This contains Supplementary Table S1-S3 and Figure S1-S3.

Table S1. Accession number of registered ACs and GCs in phylogenetic tree.

Species		Accession No.	Abbreviation	
Viridiplantae				
	Adiantum capillus-veneris	BAW35590.1	AdiantumCAPE	
	Anthoceros agrestis	LC588317 (in this study)	AnthocerosCAPE	
	Azolla filiculoides	Azfi_s0081.g038638	AzollaCAPE	
	Chara braunii	BAW35593.1	CharaCAPE	
	Chara braunii	GBG71085.1	Chara	
	Chlamydomonas reinhardtii	XP_001691280.1	Chlamydomonas-1	
	Chlamydomonas reinhardtii	PNW88988.1	Chlamydomonas-2	
	Chlorella variabilis	XP_005851659.1	Chlorella-1	
	Chlorella sorokiniana	PRW59677.1	Chlorella-2	
	Coccomyxa subellipsoidea C-169	XP_005649244.1	Coccomyxa-1	
	Coccomyxa subellipsoidea C-169	XP_005646174.1	Coccomyxa-2	
	Coleochaete orbicularis	GBSL01008739.1	ColeochaeteCAPE	
	Coleochaete orbicularis	GBSL01026055	Coleochaete	
	Cryptomeria japonica	LC588318 (in this study)	CryptomeriaCAPE cDNA	
	Cycas revoluta	LC588315 (in this study)	CycasCAPE	
	Ginkgo biloba	LC588316 (in this study)	GinkgoCAPE	
	Gonium pectorale	KXZ55438.1	Gonium	
	Klebsormidium nitens	GAQ89031.1	Klebsormidium-1	
	Klebsormidium nitens	GAQ78552.1	Klebsormidium-2	
	Marchantia polymorpha	PTQ35772.1	MarchantiaCAPE	
	Marchantia polymorpha	Mapoly0178s0022.1	Marchantia	
	Mesostigma viride	GBSK01038777.1	Mesostigma-1	
	Mesostigma viride	GBSK01037017	Mesostigma-2	
	Micractinium conductrix	PSC71265.1	Micractinium	
	Pilgerodendron uviferum	ETCJ_scaffold_2059111	PilgerodendronAC	
	Physcomitrella patens	XP_024382794.1	PhyscomitrellaCAPE	
	Salvinia cucullata	Sacu_v1.1_s0236.g026559	SalviniaCAPE	
	Selaginella moellendorffii	EFJ09864.1	SelaginellaCAPE	
	Taxodium distichum	FHST_scaffold_2061662	TaxodiumAC	
	Tetrabaena socialis	PNH10372.1	Tetrabaena	
	Trebouxia sp. A1-2	KAA6425186.1	Trebouxia	
	Volvox carteri f. *nagariensis*	XP_002949129.1	Volvox-1	
	Volvox carteri f. *nagariensis*	XP_002948497.1	Volvox-2	
Stramenopile				
	Achlya hypogyna	OQR90122.1	Achlya CAPE like	
	Chattonella antiqua	Chaan_24577_c0_g1_i1	Chattonella-C1	
	Chattonella antiqua	Chaan_24577_c0_g1_i1	Chattonella-C2	
	Ectocarpus siliculosus	CBJ26102.1	Ectocarpus-C1	
	Ectocarpus siliculosus	CBJ26102.1	Ectocarpus-C2	
	Phaeodactylum tricornutum CCAP 1055/1	XP_002177591.1	Phaeodactylum-C1	
	Phaeodactylum tricornutum CCAP 1055/1	XP_002177591.1	Phaeodactylum-C2	
	Phaeodactylum tricornutum CCAP 1055/1	XP_002186287.1	PhaeodactylumGC	(GC)
	Phytophthora cactorum	RAW37015.1	Phytophthora-1	
	Phytophthora parasitica	ETL50165.1	Phytophthora-2	
	Pythium insidiosum	GAX99050.1	Pythium-1	
	Pythium insidiosum	GAY06089.1	Pythium-2	
	Saprolegnia diclina VS20	XP_008616153.1	Saprolegnia-dCAPE like	
	Saprolegnia parasitica CBS 223.65	XP_012202270.1	Saprolegnia	
	Saprolegnia parasitica CBS 223.65	XP_012204290.1	Saprolegnia-pCAPE like	
	Thalassiosira pseudonana CCMP1335	XP_002289513.1	Thalassiosira-C1	
	Thalassiosira pseudonana CCMP1335	XP_002289513.1	Thalassiosira-C2	
	Thraustotheca clavata	OQS03511.1	Thraustotheca CAPE like	

Table S1. Accession number of registered ACs and GCs in phylogenetic tree.

Species		Accession No.	Abbreviation	
Alveolata				
	Besnoitia besnoiti	PFH34891.1	Besnoitia	
	Eimeria maxima	XP_013333510.1	Eimeria	
	Hammondia hammondi	KEP62983.1	Hammondia	
	Hepatocystis sp. ex *Piliocolobus tephrosceles*	VWU49457.1	Hepatocystis	
	Ichthyophthirius multifiliis	XP_004024096.1	Ichthyophthirius	
	Paramecium tetraurelia	CAC81657.1	Paramecium-1	
	Paramecium tetraurelia	XP_001456616.1	Paramecium-2	
	Plasmodium falciparum	CAC81938.2	Plasmodium-f	
	Plasmodium reichenowi	CDO66529.1	Plasmodium-r	
	Stentor coeruleus	OMJ78156.1	Stentor	
	Tetrahymena pyriformis	CAC81658.1	Tetrahymena	
	Toxoplasma gondii ME49	XP_002368352.1	Toxoplasma	
	Vitrella brassicaformis CCMP3155	CEM03412.1	Vitrella	
Cyanobacteria				
	Anabaena cylindrica NIES-19 PCC 7122	BAA09511.1	AnabaenacCya	
	Arthrospira platensis NIES-39	BAA22996.1	ArthrospiraCyaA	
	Arthrospira platensis NIES-39	BAA22997.1	ArthrospiraCyaC	
	Arthrospira platensis NIES-39	BAB19924.1	ArthrospiraCyaG	
	Nostoc sp. PCC 7120	BAA13997.1	NostocCyaA	
	Nostoc sp. PCC 7120	BAA13998.2	NostocCyaB1	
	Nostoc sp. PCC 7120	BAA13999.1	NostocCyaB2	
	Nostoc sp. PCC 7120	BAA14000.1	NostocCyaC	
	Nostoc sp. PCC 7120	BAA14001.1	NostocCyaD	
	Nostoc sp. PCC 7120	WP_010994837.1	NostocCyaE	
	Synechocystis sp. PCC 6803	BAA17880.1	SynechocystisCya1	
	Synechocystis sp. PCC 6803	P72951	SynechocystisCya2	
Bacteria				
	Chloroflexus aurantiacus	WP_012258114.1	Chloroflexus-C1	
	Chloroflexus aurantiacus	WP_012258114.1	Chloroflexus-C2	
	Mycobacterium tuberculosis CDC1551_sp	P9WQ34.1	Mycobacterium	
	Myxococcus xanthus	BAC00918.1	Myxococcus	
	Sinorhizobium meliloti	AAA26247.1	SinorhizobiumAC3	
Excavata				
	Euglena gracilis	BAB85619.1	EuglenaPACa-C1	
	Euglena gracilis	BAB85619.1	EuglenaPACa-C2	
	Euglena gracilis	BAB85620.1	EuglenaPACb-C1	
	Euglena gracilis	BAB85620.1	EuglenaPACb-C2	
	Euglena gracilis	BAD20740.1	EgTAC1	(Excavata AC)
	Euglena gracilis	BAD20741.1	EgTAC2	(Excavata AC)
	Leishmania donovani	AAA74998.1	Leishmania	(Excavata AC)
	Trypanosoma brucei brucei	Q99279.1	TrypanosomaAC1	(Excavata AC)
	Trypanosoma brucei brucei	AAA30158.1	TrypanosomaAC2	(Excavata AC)
	Trypanosoma brucei brucei	CAA09951.1	TrypanosomaAC3	(Excavata AC)
	Trypanosoma congolense	CAA91903.1	TcoAC	(Excavata AC)
	Trypanosoma cruzi	AAC61849.1	TcrAC1	(Excavata AC)
	Trypanosoma cruzi	CAA09737.2	TcrAC2	(Excavata AC)
	Trypanosoma equiperdum	CAA42029.1	TeAC	(Excavata AC)
Amoebozoa				
	Dictyostelium discoideum	AAA33163.1	DictyosteliumACA-C1	
	Dictyostelium discoideum	AAA33163.1	DictyosteliumACA-C2	
	Dictyostelium discoideum	AAD50121.1	DictyosteliumACB	
	Dictyostelium discoideum	AAA33164.1	DictyosteliumACG	

Table S1. Accession number of registered ACs and GCs in phylogenetic tree.

Species		Accession No.	Abbreviation	
Fungi				
	Botrytis cinerea	CAB77164.1	Botrytis	(Fungi AC)
	Candida albicans	AAG18428.1	CandidaAC	(Fungi AC)
	Cryptococcus neoformans var. grubii	AAG60619.1	CryptococcusAC	(Fungi AC)
	Lachancea kluyveri	CAA39513.1	Lachancea	(Fungi AC)
	Madurella mycetomatis	KXX82884.1	Madurella	(Fungi AC)
	Neurospora crassa	XP_011393197.1	Neurospora	(Fungi AC)
	Saccharomyces cerevisiae	CAA89295.1	Saccharomyces	(Fungi AC)
	Schizosaccharomyces pombe	AAA35284.1	Schizosaccharomyces	(Fungi AC)
	Sordaria macrospora	CAP09208.1	Sordaria	(Fungi AC)
	Ustilago maydis 521_sp	P49606.1	Ustilago	(Fungi AC)
	Valsa mali	KUI66200.1	Valsa	(Fungi AC)
Animal				
	Bos taurus	AAA79957.1	BosAC-C1	(AnimalAC-C1)
	Bos taurus	AAA79957.1	BosAC-C2	(AnimalAC-C2)
	Drosophila melanogaster	AAA28844.1	DrosophilaAC-C1	(AnimalAC-C1)
	Drosophila melanogaster	AAA28844.1	DrosophilaAC-C2	(AnimalAC-C2)
	Drosophila melanogaster	AAA74408.1	DrosophilaGC	(GC)
	Homo sapiens	NP_066939.1	HomoAC1-C1	(AnimalAC-C1)
	Homo sapiens	NP_065433.2	HomoAC2-C1	(AnimalAC-C1)
	Homo sapiens	NP_004027.2	HomoAC3-C1	(AnimalAC-C1)
	Homo sapiens	NP_640340.2	HomoAC4-C1	(AnimalAC-C1)
	Homo sapiens	NP_899200.1	HomoAC5-C1	(AnimalAC-C1)
	Homo sapiens	NP_056085.1	HomoAC6-C1	(AnimalAC-C1)
	Homo sapiens	NP_001105.1	HomoAC7-C1	(AnimalAC-C1)
	Homo sapiens	NP_001106.1	HomoAC8-C1	(AnimalAC-C1)
	Homo sapiens	NP_001107.2	HomoAC9-C1	(AnimalAC-C1)
	Homo sapiens	NP_066939.1	HomoAC1-C2	(AnimalAC-C2)
	Homo sapiens	NP_065433.2	HomoAC2-C2	(AnimalAC-C2)
	Homo sapiens	NP_004027.2	HomoAC3-C2	(AnimalAC-C2)
	Homo sapiens	NP_640340.2	HomoAC4-C2	(AnimalAC-C2)
	Homo sapiens	NP_899200.1	HomoAC5-C2	(AnimalAC-C2)
	Homo sapiens	NP_056085.1	HomoAC6-C2	(AnimalAC-C2)
	Homo sapiens	NP_001105.1	HomoAC7-C2	(AnimalAC-C2)
	Homo sapiens	NP_001106.1	HomoAC8-C2	(AnimalAC-C2)
	Homo sapiens	NP_001107.2	HomoAC9-C2	(AnimalAC-C2)
	Homo sapiens	AAF01340.1	HomoGC-A	(GC)
	Homo sapiens	NP_060887.2	Homo-sAC-C1	
	Homo sapiens	NP_060887.2	Homo-sAC-C2	
	Mus musculus	NP_033752.1	MusAC1-C1	(AnimalAC-C1)
	Mus musculus	NP_705762.2	MusAC2-C1	(AnimalAC-C1)
	Mus musculus	NP_001153008.1	MusAC3-C1	(AnimalAC-C1)
	Mus musculus	NP_536683.1	MusAC4-C1	(AnimalAC-C1)
	Mus musculus	NP_001012783.3	MusAC5-C1	(AnimalAC-C1)
	Mus musculus	AAA37182.1	MusAC6-C1	(AnimalAC-C1)
	Mus musculus	AAA57554.1	MusAC7-C1	(AnimalAC-C1)
	Mus musculus	AAB41885.1	MusAC8-C1	(AnimalAC-C1)
	Mus musculus	CAA90570.1	MusAC9-C1	(AnimalAC-C1)
	Mus musculus	NP_033752.1	MusAC1-C2	(AnimalAC-C2)
	Mus musculus	NP_705762.2	MusAC2-C2	(AnimalAC-C2)
	Mus musculus	NP_001153008.1	MusAC3-C2	(AnimalAC-C2)
	Mus musculus	NP_536683.1	MusAC4-C2	(AnimalAC-C2)
	Mus musculus	NP_001012783.3	MusAC5-C2	(AnimalAC-C2)
	Mus musculus	AAA37182.1	MusAC6-C2	(AnimalAC-C2)
	Mus musculus	AAA57554.1	MusAC7-C2	(AnimalAC-C2)
	Mus musculus	AAB41885.1	MusAC8-C2	(AnimalAC-C2)
	Mus musculus	CAA90570.1	MusAC9-C2	(AnimalAC-C2)

Table S1. Accession number of registered ACs and GCs in phylogenetic tree.

Species		Accession No.	Abbreviation	
Animal				
	Mus musculus	NP_766617.2	Mus-sAC10-1-C1	
	Mus musculus	NP_766617.2	Mus-sAC10-1-C2	
	Oryctolagus cuniculus	CAA82562.1	OryctolagusAC-C1	(AnimalAC-C1)
	Oryctolagus cuniculus	CAA82562.1	OryctolagusAC-C2	(AnimalAC-C2)
	Rattus norvegicus	AAA40682.1	RattusAC2-C1	(AnimalAC-C1)
	Rattus norvegicus	AAB39764.1	RattusAC5-C1	(AnimalAC-C1)
	Rattus norvegicus	AAA40682.1	RattusAC2-C2	(AnimalAC-C2)
	Rattus norvegicus	AAB39764.1	RattusAC5-C2	(AnimalAC-C2)
	Rattus norvegicus	AAA41201.1	RattusGC-C	(GC)
	Rattus norvegicus	AAC52417.1	RattusksGC	(GC)
	Rattus norvegicus	AAA41200.1	RattusGC-A	(GC)
	Rattus norvegicus	AAB17953.1	RattusGC-Salpha1	(GC)
	Rattus norvegicus	P20595.2	RattusGC-Sbeta1	(GC)
	Strongylocentrotus purpuratus	AAA30051.1	StrongylocentrotusGC	(GC)

Table S2. Accession number of registered PDEs in phylogenetic tree.

Species		Accession No.	Abbreviation
Viridiplantae			
	Adiantum capillus-veneris	BAW35590.1	AdiantumCAPE
	Anthoceros agrestis	LC588317 (in this study)	AnthocerosCAPE
	Azolla filiculoides	Azfi_s0081.g038638	AzollaCAPE
	Bathycoccus prasinos	XP_007512124.1	Bathycoccus
	Chara braunii	BAW35593.1	CharaCAPE
	Chlamydomonas reinhardtii	PNW87509.1	Chlamydomonas
	Chloropicon primus	QDZ24568.1	Chloropicon
	Coleochaete orbicularis	GBSL01005997.1	Coleochaete
	Coleochaete orbicularis	GBSL01008739.1	ColeochaeteCAPE
	Cryptomeria japonica	LC588319 (in this study)	CryptomeriaCAPE
	Cycas revoluta	LC588315 (in this study)	CycasCAPE
	Ginkgo biloba	LC588316 (in this study)	GinkgoCAPE
	Gonium pectorale	KXZ50318.1	Gonium
	Klebsormidium nitens	GAQ77586.1	Klebsormidium
	Marchantia polymorpha	PTQ35772.1	MarchantiaCAPE
	Mesostigma viride	GBEK01001697.1	Mesostigma1
	Mesostigma viride	GBEK01001701.1	Mesostigma2
	Mesostigma viride	GBSK01059562.1	Mesostigma3
	Mesostigma viride	GBEK01001659.1	Mesostigma4
	Ostreococcus tauri	OUS44058.1	Ostreococcus
	Physcomitrella patens	XP_024382794.1	PhyscomitrellaCAPE
	Salvinia cucullate	Sacu_v1.1_s0236.g026559	SalviniaCAPE
	Selaginella moellendorffii	EFJ09864.1	SelaginellaCAPE
	Volvox carteri f. *nagariensis*	XP_002950511.1	Volvox
Stramenopile			
	Achlya hypogyna	OQR90122.1	Achlya CAPE like
	Achlya hypogyna	OQR94169.1	Achlya
	Chattonella antiqua	Chaan_26589_c0_g1_i1_m61498	Chattonella1
	Chattonella antiqua	Chaan_24326_c0_g1_i1_m36734	Chattonella2
	Dictyopteris undulata	GFKK01054719.1	Dictyopteris
	Dinobryon sp. LO226KS	HAGC01022162.1	Dinobryon
	Ectocarpus siliculosus	CBJ33418.1	Ectocarpus
	Phytophthora kernoviae	RLN60791.1	Phytophthora1
	Phytophthora kernoviae	RLN54307.1	Phytophthora2
	Pythium oligandrum	TMW64435.1	Pythium
	Saprolegnia diclina VS20	XP_008616153.1	Saprolegnia-d CAPE like
	Saprolegnia parasitica CBS 223.65	XP_012204290.1	Saprolegnia-p CAPE like
	Saprolegnia parasitica CBS 223.65	XP_012195684.1	Saprolegnia
	Sargassum vulgare	GFKX01008947.1	Sargassum1
	Sargassum vulgare	GEHA01016282.1	Sargassum2
	Thraustotheca clavata	OQS03511.1	Thraustotheca CAPE like
	Thraustotheca clavata	OQS06861.1	Thraustotheca
	Thalassiosira oceanica	EJK58346.1	Thalassiosira-o
	Thalassiosira rotula	GGLQ01019201.1	Thalassiosira-r
Alveolata			
	Ichthyophthirius multifiliis	XP_004037103.1	Ichthyophthirius1
	Ichthyophthirius multifiliis	XP_004030322.1	Ichthyophthirius2
	Paramecium tetraurelia strain d4-2	XP_001443417.1	Paramecium
	Plasmodium falciparum 3D7	XP_001349954.1	Plasmodium1
	Plasmodium falciparum 3D7	XP_002809026.1	Plasmodium2
	Plasmodium falciparum 3D7	XP_024329151.1	Plasmodium3
	Plasmodium falciparum 3D7	XP_001348846.2	Plasmodium4
	Stentor coeruleus	OMJ67820.1	Stentor1
	Stentor coeruleus	OMJ90718.1	Stentor2
	Tetrahymena thermophila SB210	XP_001019765.3	Tetrahymena
	Theileria annulata	XP_954120.1	Theileria
Excavata			
	Naegleria fowleri	KAF0974972.1	Naegleria
	Trypanosoma cruzi	AAL58095.1	TrypanosomaPDEA
	Trypanosoma cruzi	AAM28249.1	TrypanosomaPDEB1
	Trypanosoma cruzi	XP_803815.1	TrypanosomaPDEB2

Table S2. Accession number of registered PDEs in phylogenetic tree.

Species		Accession No.	Abbreviation
Amoebozoa			
	Planoprotostelium fungivorum	PRP89163.1	Planoprotostelium
	Thecamonas trahens ATCC 50062	XP_013757491.1	Thecamonas
Fungi			
	Powellomyces hirtus	TPX62813.1	Powellomyces
	Spizellomyces punctatus DAOM BR117	XP_016607590.1	Spizellomyces
Animal			
	Danio rerio	XP_692892.6	DanioPDE1A
	Danio rerio	XP_691883.4	DanioPDE3B
	Danio rerio	XP_005165938.1	DanioPDE4B
	Danio rerio	XP_005163431.3	DanioPDE4D
	Danio rerio	NP_001116732.1	DanioPDE5A
	Danio rerio	NP_957165.1	DanioPDE6C
	Danio rerio	NP_002594.1	DanioPDE7A
	Danio rerio	XP_009293456.1	DanioPDE8B
	Danio rerio	XP_009302928.1	DanioPDE9A
	Danio rerio	AAH49532.1	DanioPDE10A
	Danio rerio	XP_697567.3	DanioPDE11
	Homo sapiens	BAB20055.1	HomoPDE1A
	Homo sapiens	NP_000915.1	HomoPDE1B
	Homo sapiens	NP_005011.1	HomoPDE1C
	Homo sapiens	AAH40974.1	HomoPDE2A
	Homo sapiens	AAB18673.1	HomoPDE3A
	Homo sapiens	NP_000913.2	HomoPDE3B
	Homo sapiens	AAU82096.1	HomoPDE4A
	Homo sapiens	NP_001032417.1	HomoPDE4B
	Homo sapiens	AAB96875.1	HomoPDE4C
	Homo sapiens	Q08499.2	HomoPDE4D
	Homo sapiens	O76074.2	HomoPDE5A
	Homo sapiens	NP_000431.2	HomoPDE6A
	Homo sapiens	P35913.2	HomoPDE6B
	Homo sapiens	P51160.2	HomoPDE6C
	Homo sapiens	NP_002594.1	HomoPDE7A
	Homo sapiens	NP_061818.1	HomoPDE7B
	Homo sapiens	NP_002596.1	HomoPDE8A
	Homo sapiens	NP_003710.1	HomoPDE8B
	Homo sapiens	NP_002597.1	HomoPDE9A
	Homo sapiens	NP_006652.1	HomoPDE10A
	Homo sapiens	NP_058649.3	HomoPDE11A

Table S3. The PDE and AC domains of gymnosperms CAPE in the OneKP database.

Family	Species	PDE*	AC*	Sequence ID
Cupressaceae	*Callitris gracilis* (taxid:214220)	○		IFLI_scaffold_2057545
	Pilgerodendron uviferum (taxid:103979)	○		ETCJ_scaffold_2051372
		○		ETCJ_scaffold_2056181
			●	ETCJ_scaffold_2059111
	Taxodium distichum (taxid:28982)		●	FHST_scaffold_2061662
	Widdringtonia cedarbergensis (taxid:13760)		○	AUDE_scaffold_2029632
			○	AUDE_scaffold_2034839
Taxaceae	*Amentotaxus argotaenia* (taxid:25625)		○	IAJW_scaffold_2135942
	Cephalotaxus harringtonia (taxid:58029)	○		GJTI_scaffold_2038034
			○	GJTI_scaffold_2046253
			○	GJTI_scaffold_2056587
		○	○	GJTI_scaffold_2061849
			○	WYAJ_scaffold_2013049
			○	WYAJ_scaffold_2031765
		○		WYAJ_scaffold_2060862
		○		WYAJ_scaffold_2082430
	Pseudotaxus chienii (taxid:89481)	○		YLPM_scaffold_2066289
	Torreya nucifera (taxid:50189)		○	HQOM_scaffold_2031209
			○	HQOM_scaffold_2114042
Zamiaceae	*Stangeria eriopus* (taxid:34343)	○		KAWQ_scaffold_2021063

*Partial (○) or full length (●) sequences homologous to PDE or AC domains of CAPE are marked.

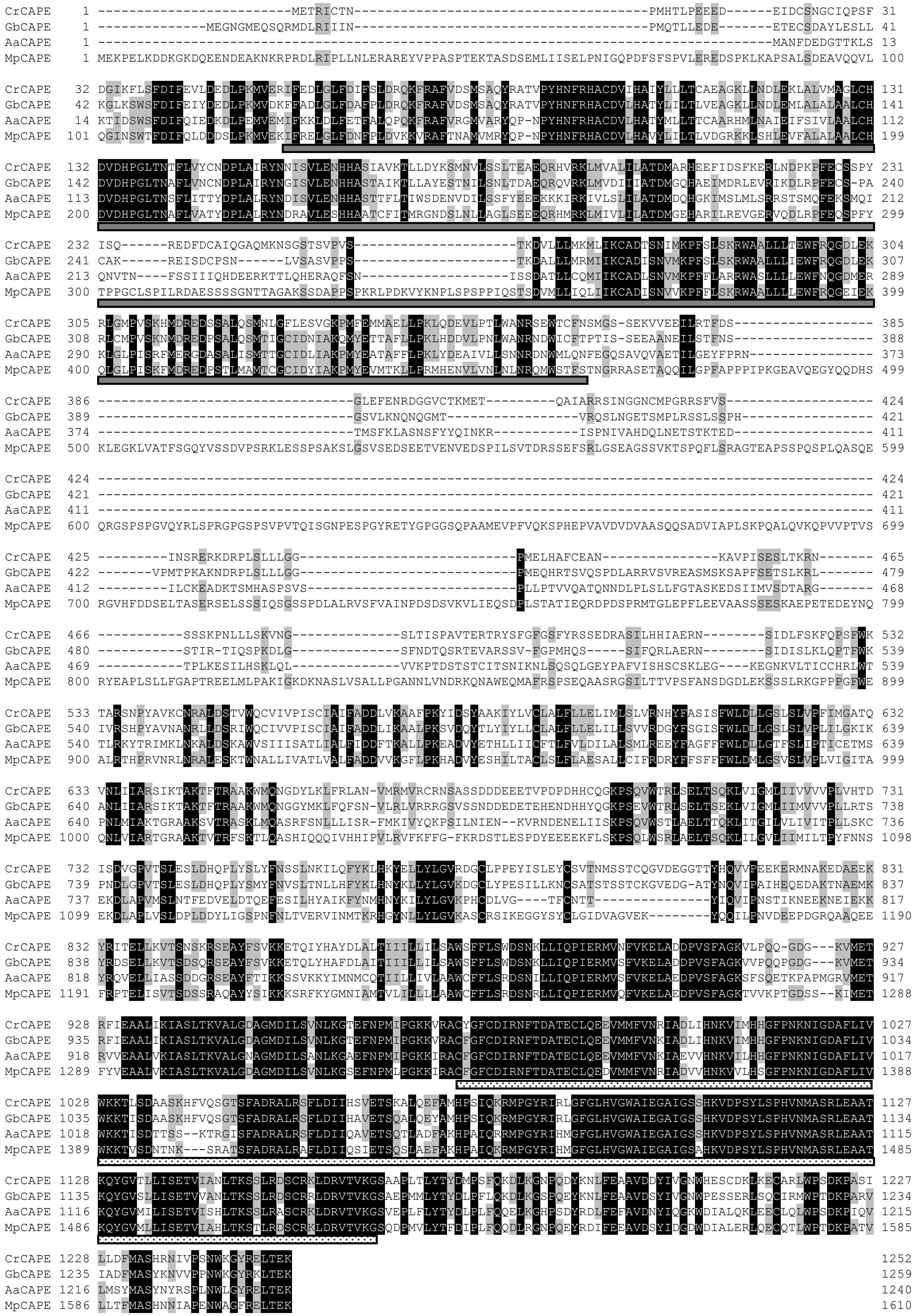

Figure S1. Multiple sequence alignment of CAPE

The amino acid sequences of CrCAPE, GbCAPE, AaCAPE, and MpCAPE are shown. Amino acid residues that were identical in the majority of the sequences are shown in gray. Fully identical amino acids in all sequences are shown in black. The catalytic domains of PDE and AC are shown as gray and dotted underlines, respectively.

Aa, *Anthoceros agrestics*; Cr, *Cycas revoluta*; Gb, *Ginkgo biloba*; Mp, *Marchantia polymorpha*

Species*	Number of transmembrane regions in CAPE**	Domain structures of CAPE***
Gymnosperms		
*Cycas revoluta**	4	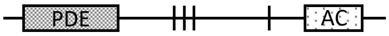
*Ginkgo biloba**	4	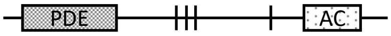
Ferns		
Adiantum capillus-veneris	4	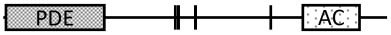
Azolla filiculoides	4	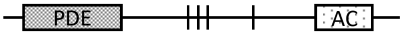
Salvinia cucullata	4	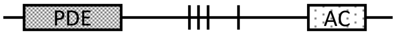
Lycophytes		
Selaginella moellendorffii	2	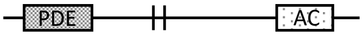
Bryophytes		
*Anthoceros agrestis**	4	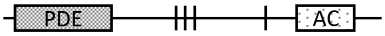
Physcomitrella patens	4	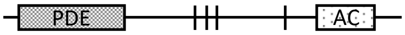
Marchantia polymorpha	2	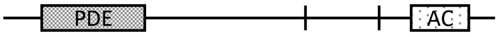
Streptophyte algae		
Chara braunii	3 (4)	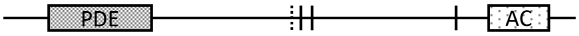
Coleochaete orbicularis	4	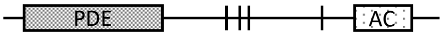

*DATA were obtained in this study.

**The transmembrane regions of CAPE were predicted with the TMHMM v2.0 program. In *Chara braunii*, the transmembrane region was predicted with the SOSUI engine ver. 1.11 program, and the predicted number was shown in parenthesis.

***The transmembrane regions are shown as vertical lines. In *Chara braunii*, the additional transmembrane region by SOSUI program is shown as a dotted vertical line.

Figure S2. Number of transmembrane regions and domain structures of CAPE

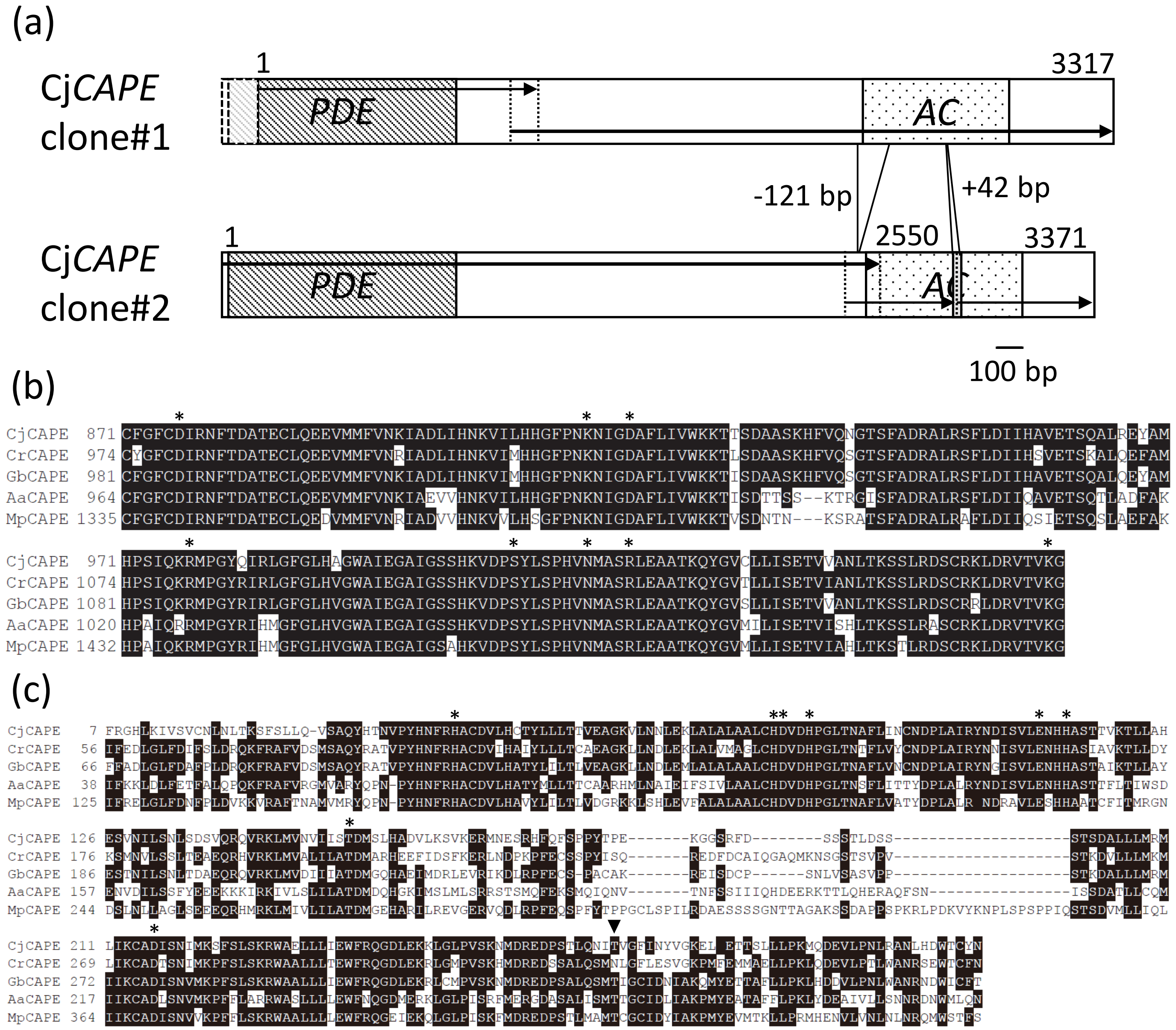

Figure S3. CAPE cDNA clones obtained from *Cryptomeria japonica*

(a) Schematic representation of Cj*CAPE*. Cj*CAPE* clone#1 encoded a partial PDE domain that lacks 14 amino acids at the N-terminus and complete AC domain. Cj*CAPE* clone#2 encoded PDE domain and an incomplete AC domain caused by the deletion of 121 bp just before the AC domain and insertion of 42 bp at the middle of the AC-coding sequence. Open reading frames are shown by arrows.

(b) Multiple alignment of the AC domain of CAPE. Amino acids involved in binding the substrate ATP are indicated by asterisks. Amino acid residues that were identical in the majority of the sequences are shown in black. The amino acids sequence for CjCAPE was used from clone#1.

(c) Multiple alignment of the PDE domain of CAPE. Amino acids involved in metal ion binding are indicated by asterisks. Arrowhead shows amino acid residues involved in the recognition of purine bases. Amino acid residues that were identical in the majority of the sequences are shown in black. The amino acids sequence for CjCAPE was used from clone#2.

Aa, *Anthoceros agrestis*; Cj, *Cryptomeria japonica*; Cr, *Cycas revoluta*; Gb, *Ginkgo biloba*; Mp, *Marchantia polymorpha*
